# Overexpression of a citrus NDR1 ortholog increases disease resistance in *Arabidopsis*

**DOI:** 10.3389/fpls.2013.00157

**Published:** 2013-06-03

**Authors:** Hua Lu, Chong Zhang, Ute Albrecht, Rena Shimizu, Guanfeng Wang, Kim D. Bowman

**Affiliations:** ^1^Department of Biological Sciences, University of Maryland Baltimore CountyBaltimore, MD, USA; ^2^United States Horticultural Research Laboratory, Agricultural Research Service, United States Department of AgricultureFort Pierce, FL, USA

**Keywords:** *Pseudomonas syringae*, salicylic acid, citrus canker, Huanglongbing, greening disease, *Candidatus* Liberibacter asiaticus, genetic engineering

## Abstract

Emerging devastating diseases, such as Huanglongbing (HLB) and citrus canker, have caused tremendous losses to the citrus industry worldwide. Genetic engineering is a powerful approach that could allow us to increase citrus resistance against these diseases. The key to the success of this approach relies on a thorough understanding of defense mechanisms of citrus. Studies of *Arabidopsis* and other plants have provided a framework for us to better understand defense mechanisms of citrus. Salicylic acid (SA) is a key signaling molecule involved in basal defense and resistance (R) gene-mediated defense against broad-spectrum pathogens. The *Arabidopsis* gene *NDR1* (NON-RACE-SPECIFIC DISEASE RESISTANCE 1) is a positive regulator of SA accumulation and is specifically required for signaling mediated by a subset of R genes upon recognition of their cognate pathogen effectors. Our bioinformatic analysis identified an ortholog of *NDR1* from citrus, *CsNDR1*. Overexpression of *CsNDR1* complemented susceptibility conferred by the *Arabidopsis* *ndr1-1 *mutant to *Pseudomonas syringae* strains and also led to enhanced resistance to an oomycete pathogen *Hyaloperonospora arabidopsidis*. Such heightened resistance is associated with increased SA production and expression of the defense marker gene *PATHOGENESIS RELATED 1* (*PR1*). In addition, we found that expression of *PR1* and accumulation of SA were induced to modest levels in citrus infected with *Candidatus* Liberibacter asiaticus, the bacterial pathogen associated with HLB disease. Thus, our data suggest that *CsNDR1* is a functional ortholog of *Arabidopsis* *NDR1*. Since *Ca.* L. asiaticus infection only activates modest levels of defense responses in citrus, we propose that genetically increasing SA/NDR1-mediated pathways could potentially lead to enhanced resistance against HLB, citrus canker, and other destructive diseases challenging global citrus production.

## INTRODUCTION

Huanglongbing (HLB; also called citrus greening disease), citrus canker, and other emerging diseases have imposed serious threats to the citrus industry worldwide (Bove, 2006; Gottwald, 2007). Citrus canker is caused by the gram negative bacterium *Xanthomonas axonopodis* pv.* citri*. Wind and rain facilitate the dispersal of the pathogen and spreading of the disease. More than 16 million citrus trees have been destroyed in Florida in an effort to restrict the disease (Gaskalla, 2006). HLB is even more devastating than citrus canker since it is highly contagious and lethal to afflicted plants (Bove, 2006; Brlansky and Rogers, 2007; Gottwald, 2007). The parasitic bacterium *Candidatus* Liberibacter that lives in the phloem tissue of a citrus tree is believed to be the associating agent of HLB. The disease is transmitted by a small insect vector of the family Psyllidae. The growth of psyllids cannot yet be reliably controlled by conventional insecticide application (Ichinose et al., 2010). Without successful measures to control the causal pathogen and its transmission vector, HLB is endemic to a variety of citrus species and related plants. Therefore, it is imperative to develop strategies to contain and eventually eradicate HLB and other diseases challenging the production of citrus worldwide.

Successful manipulation of citrus disease resistance relies on a thorough understanding of defense mechanisms of the plant. Although not well understood yet in citrus, defense mechanisms are well studied in other plants, in particular the model plant *Arabidopsis thaliana*, and have been shown to be relatively conserved among plants (Nishimura and Dangl, 2010). Therefore, prior knowledge of defense mechanisms from other plants should help us to better understand citrus defense and ultimately develop effective strategies to combat devastating diseases challenging the citrus industry.

Plant defense can be preformed or induced. The preformed defense includes some existing physical structures and chemical compounds produced by plants before infection that can restrict pathogen invasion. The induced defense can be activated upon pathogen invasion, involving sophisticated surveillance systems to recognize general elicitors from pathogens and subsequently to activate basal defense. Much stronger defense can be induced when host resistance (R) proteins specifically recognize their cognate pathogen effectors; thus such defense is also termed as R protein-mediated defense. The largest class of R proteins is represented by a family of proteins that have two conserved domains, nucleotide binding site (NBS) and leucine-rich repeat (LRR; Martin et al., 2003; McDowell and Simon, 2006). This class of R proteins can be further divided into two groups according to the N-terminal sequence, coiled-coil (CC)–NBS–LRR and Toll-interleukin-1 receptor (TIR)–NBS–LRR. Some CC–NBS–LRR type proteins are found to signal through NON-RACE-SPECIFIC DISEASE RESISTANCE 1 (NDR1). For instance, an *ndr1* mutation compromises resistance conferred by the CC–NBS–LRR proteins RPS2, RPM1, or RPS5 to *Pseudomonas syringae* expressing the avirulence effectors *avrRpt2*, *avrB* and *avrRpm1*, or *avrPph3*, respectively (Century et al., 1995; Aarts et al., 1998). On the other hand, some TIR–NBS–LRR type proteins functionally require ENHANCED DISEASE SUSCEPTIBILITY 1 (EDS1). For instance, an *eds1* mutant is immune-compromised to *P. syringae avrRps4*, resistance to which is conferred by the TIR–NBS–LRR protein RPS4 but not by TIR–NBS–LRR type R proteins (Aarts et al., 1998; Falk et al., 1999). These observations suggest a general rule that these two subgroups of R proteins can activate distinct downstream signaling pathways. However, exceptions to this rule also exist for some other NBS–LRR R proteins (McDowell et al., 2000; Bittner-Eddy and Beynon, 2001; Xiao et al., 2001).

The small phenolic molecule salicylic acid (SA) plays a key role in signaling both basal and R protein-mediated defense (Hammond-Kosack and Jones, 1996; Tsuda et al., 2008) and is involved in resistance against diverse pathogens and in response to various stress conditions (Malamy et al., 1990; Rassmussen et al., 1991; Sharma and Davis, 1997; Tsuda et al., 2008). While increased SA accumulation and/or signaling lead to enhanced disease resistance, disrupting these processes by gene mutations or transgene expression result in compromised defense against pathogens (Durrant and Dong, 2004; Lu, 2009). Genes involved in SA-mediated defense can affect SA biosynthesis, accumulation, and/or signaling (Lu, 2009). For instance, *SA INDUCTION-DEFICIENT 2 EDS16*, encodes isochorismate synthase contributing to the majority of SA biosynthesis (Wildermuth et al., 2001). Both *NDR1* and *EDS1* are known to act upstream of SA to regulate SA accumulation (Falk et al., 1999; Shapiro and Zhang, 2001). Downstream of SA signaling, *NONEXPRESSOR OF PR GENES 1 NPR1*) acts as a signal transducer that regulates systemic acquired resistance, a long-lasting defense against broad-spectrum pathogens at the whole plant level (Cao et al., 1997; Ryals et al., 1997; Shah et al., 1997; Dong, 2004). Overexpression of *NDR1*, *EDS1*, *NPR1*, or several other SA regulators confers enhanced disease resistance to a range of pathogens in *Arabidopsis* and/or in other plants (Chern et al., 2001; Fitzgerald et al., 2004; Lin et al., 2004; Makandar et al., 2006; Malnoy et al., 2007; Pegadaraju et al., 2007; Sandhu et al., 2009; Gao et al., 2010). Therefore, manipulation of SA-mediated defense has the potential to introduce broad-spectrum disease resistance in plants.

*NDR1* encodes a glycosyl-phosphatidyl inositol-anchored plasma membrane protein that belongs to a large protein family (Dormann et al., 1995; Varet et al., 2002; Coppinger et al., 2004; Zheng et al., 2004). A recent study implicates the function of *NDR1* in mediating plasma membrane-cell wall adhesion (Knepper et al., 2011). *NDR1-*like genes widely exist in different plants (Lee et al., 2006; Chong et al., 2008; Cacas et al., 2011). Besides *NDR1*, some *Arabidopsis* homologs of *NDR1* were shown to be highly induced by pathogen infection and/or to confer enhanced disease resistance to *P. syringae* when overexpressed (Varet et al., 2002, 2003; Coppinger et al., 2004; Zheng et al., 2004). Thus *NDR1* and some members in the family are critical components of plant defense.

In this study, we report the isolation and characterization of a functional ortholog of *NDR1* in citrus, named *CsNDR1*. We found that overexpression of *CsNDR1* complements the susceptibility of *Arabidopsis* *ndr1-1* mutant to *P. syringae avrRpt2* and further confers enhanced disease resistance to *P. syringae avrRps4*, which normally is not affected by the endogenous *NDR1*. Overexpression of *CsNDR1* also led to increased resistance to the oomycete pathogen *Hyaloperonospora arabidopsidis *(*Hpa*) isolate Noco2. *CsNDR1*-induced disease resistance is associated with increased SA accumulation and expression of the defense marker gene *PATHOGENESIS RELATED 1* (*PR1*) in the transgenic *Arabidopsis* plants. In addition, we found that citrus infected with *Candidatus* Liberibacter asiaticus, a pathogen associated with the HLB disease, expressed modestly increased *CsNDR1* and SA levels, compared with mock-treated plants. We propose that genetic engineering to enhance SA/NDR1 signaling pathway in citrus could potentially enhance its resistance to HLB, citrus canker, and other emerging diseases.

## MATERIALS AND METHODS

### PLANT MATERIALS

*Arabidopsis* plants were grown in growth chambers with a 12 h light/12 h dark cycle, light intensity at 200 μmol m^-^^2^ s^-^^1^, 60% humidity, and 22°C. The *ndr1-1* mutant was previously described (Century et al., 1995, 1997). Citrus plants, “Valencia” (*Citrus sinensis* [L.] Osbeck), were grown on the greenhouse bench and kept at 24°C under natural light conditions. Plants were irrigated as needed and fertilized every 3 weeks using a water-soluble fertilizer mix, 20N–10P–20K (Peters Professional, The Scotts Company, Marysville, OH, USA).

### BIOINFORMATIC ANALYSIS

Basic Local Alignment Search Tools (BLAST) was used to search protein sequence databases for *Arabidopsis*^1^ and *Citrus sinensis*^2^, using appropriate query sequences. Sequence alignment and phylogenetic analysis were performed with the MEGA program (version 5.05). To construct the phylogenetic tree, the neighbor-joining method with 1000 bootstrap replications was used.

### PATHOGEN INFECTIONS

*Pseudomonas syringae* strains used in this study were previously described (Lee et al., 2008; Wang et al., 2011b). Bacteria were cultured at 28°C with King’s B medium (10 g proteose peptone, 1.5 g K_2_HPO_4_, 3.2 ml 1 M MgSO_4_, and 5 g glycerol/l) containing the appropriate antibiotics. Freshly cultured bacteria at the optical density of 0.5–0.8 were harvested, washed once, and resuspended in 10 mM MgSO_4_ to make the infection solution at the desired concentrations. The fifth to seventh leaves of 25-day-old *Arabidopsis* plants were infected by infiltration with a 1-ml needleless syringe. For bacterial growth assay, six leaves selected from over 10 plants of each genotype were collected 3 days post-infiltration, bored with a core borer (6 mm in diameter), and ground for bacterial growth measurement as described previously (Lu et al., 2003). For hypersensitive response (HR) test, one-half of a leaf at the fifth, sixth, or seventh position was infiltrated with *P. syringae* pv. *maculicola* ES4326 *avrRpt2* (*Pma avrRpt2*; OD_600_ = 0.1) and scored 16–24 h post-infiltration for leaf collapse. Leaves infiltrated with 10 mM MgSO_4_ or the isogenic virulent strain *Pma* (OD_600_ = 0.1) were used as controls. At least 16 leaves from different plants of each genotype were scored for the HR symptoms. The rate of HR was expressed by the percentile of the number of leaves that developed HR symptoms out of the total number of inoculated leaves.

*Hyaloperonospora arabidopsidis* isolate Noco2 was a kind gift from S. Xiao at University of Maryland College Park. Strain propagation and preparation were conducted as previously described (Song et al., 2004; McDowell et al., 2010). *Hpa Noco2* spores (5 × 10^4^ spores/ml in water) were sprayed on 7-day-old soil-grown seedlings. Sporangiophores on both sides of cotyledons were counted 7 days post-inoculation. At least 50 cotyledons from each genotype were counted to derive the average number of sporangiophores per genotype.

For *Ca.* L. asiaticus infection, 15-month-old “Valencia” plants were inoculated by grafting with two bark- or bud-pieces and two leaf pieces from infected greenhouse-grown “Valencia” plants, which were tested PCR-positive for *Ca.* L. asiaticus and demonstrated symptoms for HLB. Plants similarly inoculated but with disease-free tissue pieces obtained from healthy greenhouse-grown “Valencia” plants were used as controls. Plants were pruned immediately after graft-inoculation to promote new leaf growth and HLB disease development. The inoculated plants were randomized periodically on the greenhouse bench to minimize the effect of environment on their defense responses to *Ca.* L. asiaticus.

### PCR-DETECTION OF *Ca.* L. asiaticus IN CITRUS

*Ca.* L. asiaticus-infected “Valencia” plants began to show typical HLB symptoms, yellowing and blotchy mottling around 11 weeks after inoculation (wai), which progressed more severely later. The symptomatic leaves were collected at 11 and 16 wai and extracted for DNA followed by PCR-detection of *Ca.* L. asiaticus (Albrecht and Bowman, 2012). Specifically, 100 mg leaf tissue was ground for DNA extraction, using the Plant DNeasy Mini Kit (Qiagen, Valencia, CA, USA) according to the manufacturer’s instructions. For detection of *Ca.* L. asiaticus, real-time PCR assays were performed using primers HLBas and HLBr and the probe HLBp as described (Li et al., 2006). Amplifications were performed over 40 cycles using an ABI 7500 real-time PCR system (Applied Biosystems, Foster City, CA, USA) and the QuantiTect Probe PCR Kit (Qiagen) according to the manufacturer’s instructions. All reactions were carried out in a 20-μl reaction volume using 5 μl DNA. Once a branch was confirmed positive for *Ca.* L. asiaticus, symptomatic leaves on the branch were harvested for further RNA and SA analyses.

### RNA EXTRACTION AND ANALYSIS

Twenty-five-day-old *Arabidopsis* plants were harvested for RNA extraction and northern blotting as described (Ng et al., 2011). Radioactive probes were made by PCR with an antisense primer specific for a gene fragment in the presence of [32P] dCTP. Primers used for making the *CsNDR1* probe were CsNDR1-F1 (ATGTCAGAAAACGCCGGTG) and CsNDR1-R1 (TTAAGCAAAAATCAAGACAAAAAAATAC). Primers for the *PR1* and *18S* *rRNA* probes were described previously (Ng et al., 2011).

Fully expanded leaves from *Ca.* L. asiaticus-infected “Valencia” plants showing HLB symptoms were collected 16 wai. One gram leaf tissue was ground in liquid nitrogen with a mortar and pestle and resuspended in 10 ml guanidinium isothiocyanate buffer (Chomczynski and Sacci, 1987). Total RNA was extracted as previously described (Strommer et al., 1993) with slight modifications. Phenol/chloroform/isoamyl alcohol (25:24:1) extraction was followed by two extractions with chloroform/isoamyl alcohol and precipitation of RNA with isopropanol at -20°C overnight. RNA was pelleted by centrifugation at 10,000 *g* and 4°C for 1 h, resuspended in 5 ml water and precipitated overnight at 0°C with an equal volume of 8 M LiCl. After centrifugation at 10,000 *g* and 4°C for 1 h, RNA was washed twice with 70% ethanol, air-dried, and dissolved in 500 μl of water. RNA was further purified, using the RNeasy^®^ MinElute Cleanup kit (Qiagen) according to the manufacturer’s instructions. Total RNA was DNase-treated using the TURBO DNA-*free*-Kit^ TM^ (Ambion, Austin, TX, USA) according to the manufacturer’s instructions.

Quantitative reverse transcription real-time PCR (qRT-PCR) was performed using an ABI 7500 real-time PCR system (Applied Biosystems) and the QuantiTect SYBR Green RT-PCR Kit (Qiagen) according to the manufacturer’s instructions. Sixty nanograms of DNase-treated RNA were used in a total volume of 20 μl. For detection of *CsNDR1* transcripts, forward primer 5′-TGCTGCAGCTTCATCTTCAC-3′ and reverse primer 5′-TGTCGTGTTGTTTCGGTTGT-3′ were used. For detection of *18S rRNA* transcripts, forward primer 5′-GCTTAGGCCAAGGAAGTTTG-3′ and reverse primer 5′-TCTATCCCCATCACGATGAA-3′ were used. Melting curve analysis was performed to ensure amplification of a single product and the absence of primer-dimers. For relative quantification of gene expression, the 2^-^^Δ^^Δ^^CT^ method was applied as previously described (Livak and Schmittgen, 2001), using cycle threshold (Ct) values of *18S rRNA* for normalization.

### cDNA AMPLIFICATION, DNA CONSTRUCTION, AND PLANT TRANSFORMATION

To obtain the full-length *CsNDR1* cDNA sequence, we used the SMARTer^ TM^ RACE cDNA Amplification Kit (Clontech) to make a cDNA library from RNA extracted from *Ca.* L. asiaticus-infected 15-month-old “Valencia” plants. Nested primers, NDR1-5R-P1 (CACTTTCTGATCGGTCAGCGCAG) and NDR1-5R-P2 (CAATCACGGACGTGCCGATG), were used to amplify the 5′ end missing sequence while NDR1-3R-P1 (CATCGGCACGTCCGTGATTG) and NDR1-3R-P2 (CTGCGCTGACCGATCAGAAAGTG) were used to amplify the 3′ end missing sequence. The amplified fragments were cloned in the pJET cloning vector, using the CloneJET^ TM^ PCR Cloning Kit (Thermo Scientific), and sequenced to obtain the full-length cDNA sequence. The full-length *CsNDR1* cDNA was further amplified from the library with NDR1-F1 (ATGTCAGAAAACGCCGGTG) and NDR1-R1 (TTAAGCAAAAATCAAGACAAAAAAATAC) and cloned into the *pJET* vector. At least 10 individual colonies were prepared for DNA and analyzed by sequencing. The sequence with fewer polymorphisms, compared with the reference sequence, and a correct open reading frame was used as the template for further cloning into the binary vector *pBINplusARS* under the control of the *CAMV 35s* promoter. The construct was confirmed by sequencing and transferred to *Agrobacterium tumefaciens* for *ndr1-1* transformation, using the floral dipping method (Clough and Bent, 1998). T_0_ seeds were selected for T_1_ plants on MS plates containing kanamycin, resistance to which was conferred by the binary vector. T_1_ transgenic plants were collected for seeds, which were further selected for homozygous T_2_ plants.

### ION LEAKAGE MEASUREMENT

Leaves of 25-day-old *Arabidopsis* plants were infiltrated with a bacterial suspension *Pma avrRpt2* (OD_600_ = 0.1) with a blunt-end syringe, using 10 mM MgSO_4_ treatment as a control. At 0, 4, and 7 h post-inoculation, five leaf disks, cut with a 6-mm core borer, were collected, washed in de-ionized water, and placed in a 15-ml tube with 5 ml of de-ionized water. Triplicate tubes for each sample were gently shaken for 15 min followed by the measurement of solution conductivity, using an electrical conductivity meter (The London Company, Welwyn International Inc. Cleveland, OH, USA). Each tube was measured three times to derive average conductivity.

### SA MEASUREMENT

Free and total SA (glucosylated SA) were extracted from 25-day-old Arabidopsis plants (Ng et al., 2011; Wang et al., 2011a). The same protocol was used to extract SA from leaves of *Ca.* L. asiaticus-positive “Valencia” plants that demonstrated HLB symptoms. SA separation and detection were conducted with a high-performance liquid chromatography (HPLC) instrument as previously described (Ng et al., 2011; Wang et al., 2011a).

## RESULTS

### IDENTIFICATION OF A CITRUS NDR1 ORTHOLOG

**FIGURE 1:**
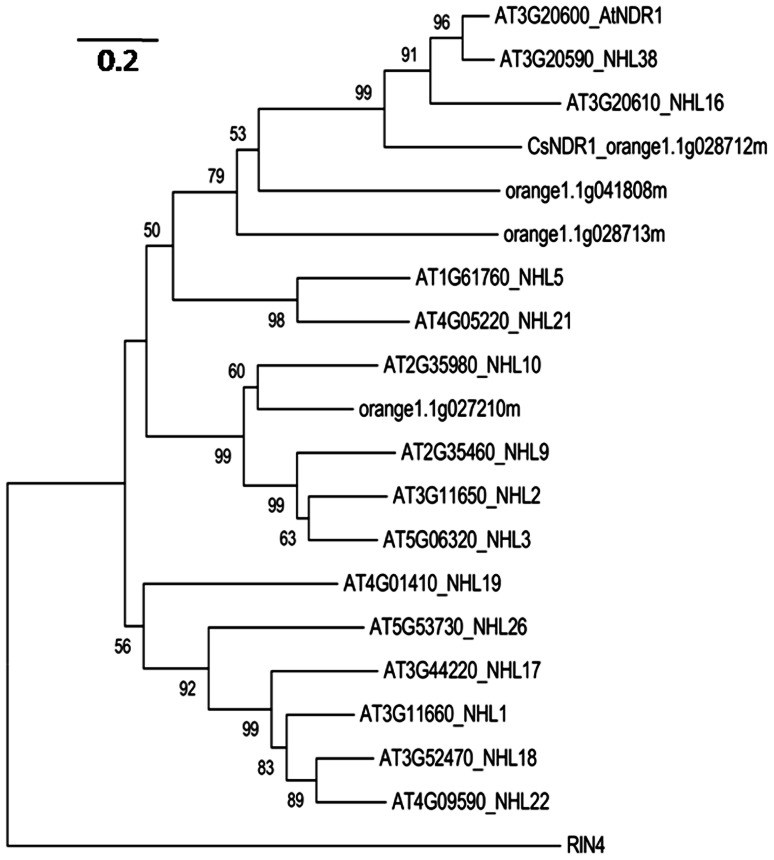
**Neighbor-joining phylogenetic tree to show relationship among NDR1 homologs from *Arabidopsis* and citrus.** The protein sequences were retrieved from BLAST search of *Citrus sinensis* protein database with NDR1 sequence as a query or from BLAST search of *Arabidopsis* protein database with CsNDR1 sequence as a query, using an *E*-value cutoff e^-^^4^. The MEGA program (version 5.05) was used to construct the tree. Protein sequences of the indicated genes were aligned with the ClustalW method and the tree was generated with the neighbor-joining method, using 1000 bootstrap replications. Numbers on the tree indicate bootstrap support (values <50% not shown). Branch lengths were drawn to scale; size bar represents number of amino acid substitutions per site. RIN4 protein sequence was used as an outgroup to root the tree.

Since NDR1 plays a critical role in *Arabidopsis* defense, we set out to identify the citrus ortholog and investigate its role in defense regulation. In *Arabidopsis*, NDR1 belongs to a large protein family with over 40 members, named NDR1/HIN1-like (NHL) proteins (Dörmanna et al., 2000). BLAST searching of the sequence database of *Citrus sinensis*^3^ with the NDR1 protein sequence revealed a citrus protein (CsNDR1; orange1.1g028712m) with the highest similarity to NDR1 (the *E*-value is 2.4e^-^^53^) and three other top hits with *E*-values below 1.0 e^-^^5^. We further used CsNDR1 as the query to search the *Arabidopsis* protein database and retrieved sequences of NDR1 and 14 NHL proteins as the top hits. To determine the extent of similarity among these proteins, phylogenetic analysis was conducted, using the MEGA program (version 5.0; Tamura et al., 2011). **Figure 1** shows that CsNDR1 is in the same cluster with NDR1 with 99% bootstrap support. Two other citrus NHL proteins (orange1.1g041808m and orange1.1g08713m) are also in the same cluster with NDR1 but with lower confidence levels in bootstrap support. Thus, bioinformatic analysis suggests that CsNDR1 is an ortholog of NDR1.

### ECTOPIC EXPRESSION OF *CsNDR1* COMPLEMENTS *Arabidopsis ndr1-1* MUTANT

**FIGURE 2:**
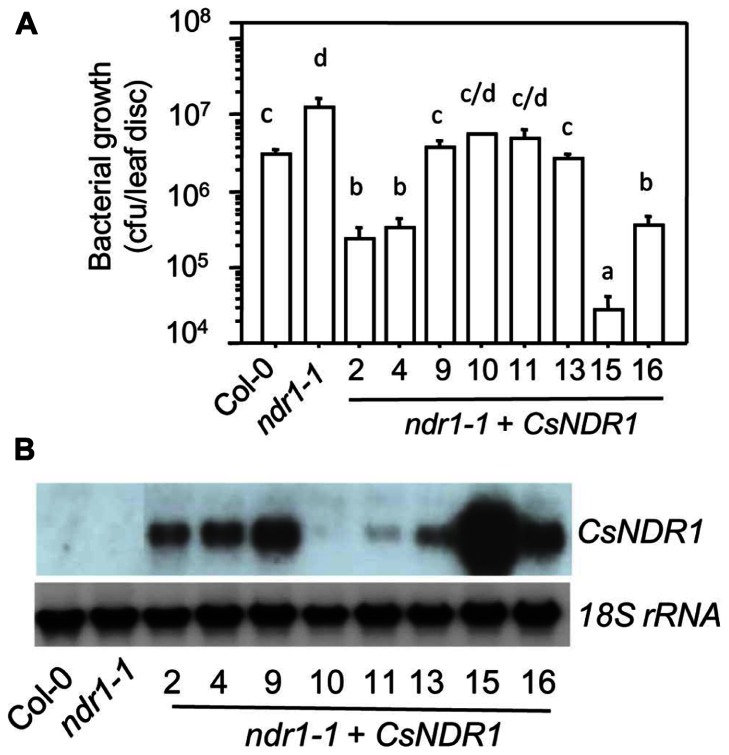
***CsNDR1* rescues enhanced disease susceptibility conferred by *Arabidopsisndr1-1 *to a virulent *P. syringae *strain. (A)** Bacterial growth assay. Twenty-five-day-old plants were infected with the virulent strain *P. syringae *pv. *maculicola* ES4326 (*Pma*; OD_600_ = 0.0001). Bacterial growth was assessed 3 days after infection. Data represent the average of bacterial numbers in six samples ± standard error. Statistical analysis was performed with log transformed data, using one-way analysis of variance (ANOVA) Fisher’s protected least significant difference (PLSD) tests (StatView 5.0.1). Different letters indicate significant difference among the samples (*P* < 0.05). **(B)** Expression analysis of transgenic plants carrying *CsNDR1*. Total RNA was extracted from 25-day-old plants and analyzed by northern blotting. A probe specific to *CsNDR1* was used to detect expression of the transgene. The *18S rRNA* probe was used to indicate equal loading in the samples. These experiments were repeated two times with similar results.

To test if *CsNDR1* shares conserved function with its *Arabidopsis* correspondence, we used a genetic complementation approach. The full-length *CsNDR1* cDNA was amplified via RT-PCR from a cDNA library made from *Ca.* L. asiaticus-infected “Valencia” plants and was cloned initially to the pJET vector and then to the binary vector *pBINplusARS* under the control of the CAMV 35s promoter. The *CsNDR1*/*pBINplusARS* construct was used to transform *ndr1-1* via the standard floral dipping method (Clough and Bent, 1998). The presence of the transgene was confirmed by PCR with gene-specific primers. Initial infection of the T_1_ transgenic plants with a virulent strain *P. syringae* pv. *maculicola* ES4326 (*Pma*) indicated that some of the transgenic plants were more resistant than *ndr1-1* (data not shown)*.* We further isolated homozygous plants for eight independently transformed lines (*ndr1-1* + *CsNDR1)*. Infection of these plants with *Pma* showed that all lines were more resistant than *ndr1-1* and some were even more resistant than Col-0 (**Figure 2A**). Total RNA was isolated from these plants and northern blotting indicated that the level of disease resistance in some transgenic plants was correlated with the degree of transgene expression (**Figure 2B**). Thus, our results suggest that *CsNDR1* positively regulates *Arabidopsis* defense.

**FIGURE 3:**
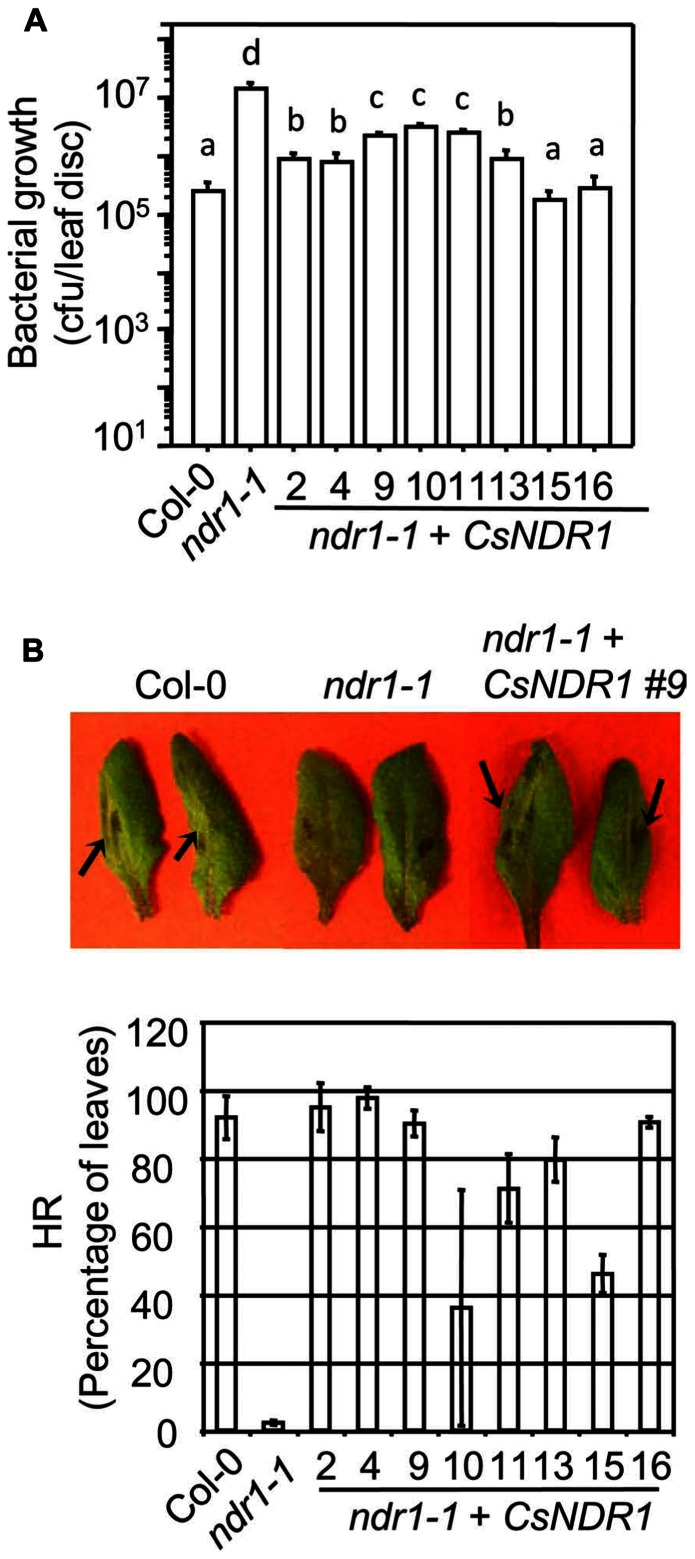
***CsNDR1* rescues enhance disease susceptibility and HR-defect conferred by *ndr1-1 *in response to *Pma avrRpt2*. (A)** Bacterial growth assay. Twenty-five-day-old plants were infected with *Pma avrRpt2* (OD_600_ = 0.0004). Bacterial growth was assessed 3 days after infection. Data represent the average of bacterial numbers in six samples ± standard error. Statistical analysis was performed with log transformed data, using one-way ANOVA Fisher’s PLSD tests (StatView 5.0.1). Different letters indicate significant difference among the samples (*P* < 0.05). **(B)** HR test. Half leaves of the fourth to seventh leaves of 25-day-old plants were infiltrated with *Pma avrRpt2* (OD_600_ = 0.1) and scored for the HR symptoms 16 h post-infection. At least 16 leaves were infected in each sample. *Top panel*: picture of infected leaves. The arrowheads indicate leaf collapse, a typical HR symptom, in Col-0 and a representative complemented plant (line 9) but not in *ndr1-1*. *Bottom panel*: the percentage of the infected leaves of each genotype that showed HR. These experiments were repeated two times with similar results.

The *Arabidopsis* RPS2 is a CC–NBS–LRR type R protein. When recognizing the avirulent strain *Pma avrRpt2*, RPS2 activates strong defense responses. Such defense activation requires the function of NDR1 and sometimes leads to HR, a rapid programed cell death in the infected region (Aarts et al., 1998). We found that all transgenic plants showed enhanced disease resistance to *Pma avrRpt2* (OD_600_ = 0.0004), compared with *ndr1-1* (**Figure 3A**). In addition, the *ndr1-1* mutant showed compromised HR in response to a high dose of *Pma avrRpt2* (OD_600_ = 0.1), as indicated by the lack of leaf collapse (**Figure 3B**, top panel; Century et al., 1995). We found that all transgenic plants showed partial to full rescue of HR-defect of *ndr1-1* (**Figure 3B**, bottom panel).

**FIGURE 4:**
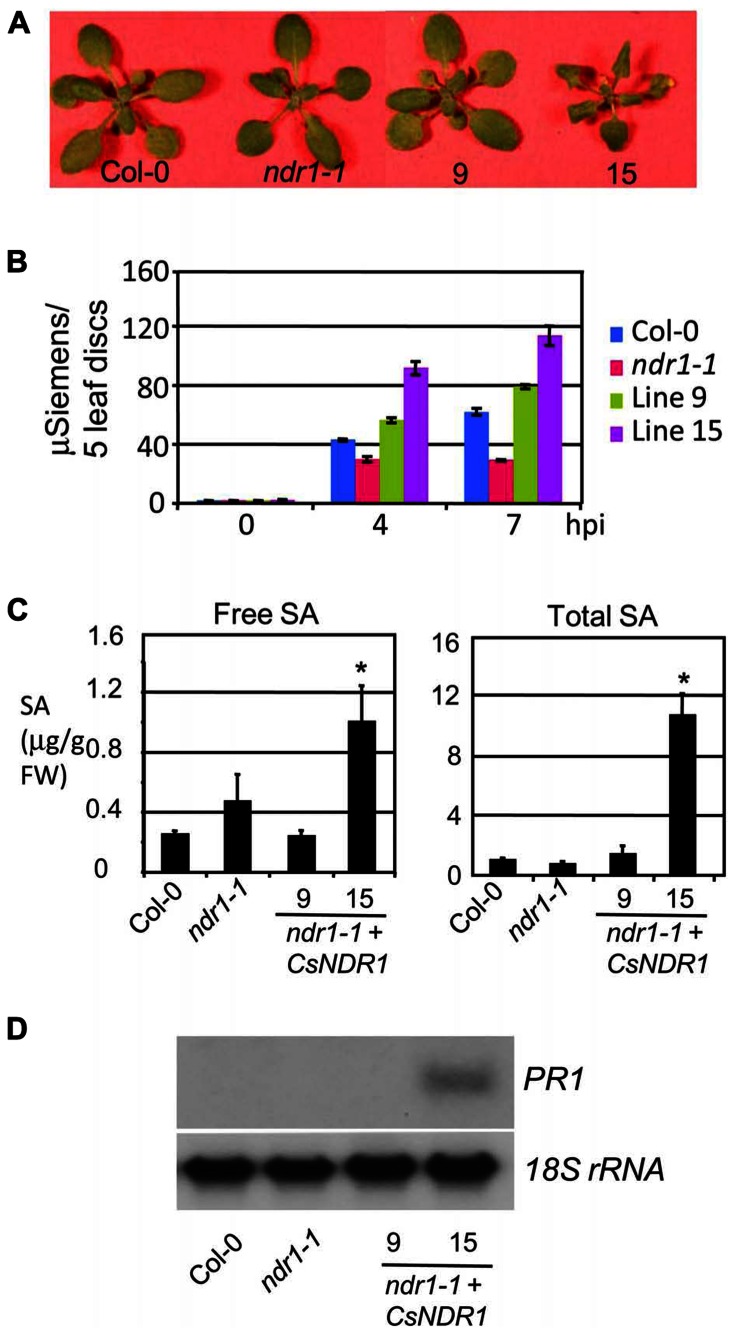
***CsNDR1* overexpression leads to increased ion leakage, SA accumulation, and *PR1* expression. (A)** Picture of 25-day-old plants. Other transgenic lines resemble Col-0 (not shown). **(B)** Ion leakage measurement. The fourth to seventh leaves of 25-day-old plants were infiltrated with *Pma avrRpt2* (OD_600_ = 0.1) and collected at the indicated times post-infection for ion leakage measurement. The data represent average of triplicate samples, each of which contained five individual leaf disks of 6 mm in diameter. **(C)** SA quantitation. Twenty-five-day-old plants were harvested for SA extraction followed by HPLC analysis. Asterisks indicate significant difference between the samples and Col-0 (*P* < 0.05; *n* = 3). **(D)** Northern blotting. DNA fragments specific to *PR1* and *18s rRNA*, respectively, were used to probe blots containing RNA samples from Col-0, *ndr1-1*, and transgenic lines 9 and 15. These experiments were repeated two times with similar results.

Interestingly, line 15 that showed the highest level of *CsNDR1* expression, had a low frequency of leaf collapse in the HR assay, albeit still more than the *ndr1-1* mutant. We also noticed that this line is smaller than other lines (**Figure 4A**). Thus we suspected that the small leaf size might obscure the HR scoring. To better quantify the HR cell death, we performed ion leakage measurement with this line and another line (#9) that showed medium *CsNDR1* expression (**Figure 2B**). When challenged with *Pma avrRpt2 *(OD_600_ = 0.1), *ndr1-1* had the lowest level of ion leakage (**Figure 4B**; Zhang et al., 2004), consistent with its HR-deficit. The ion leakage level was highest in line 15 and medium in line 9, compared with Col-0. Together disease resistance and HR assays suggest that *CsNDR1* functions similarly as *Arabidopsis* *NDR1* in both basal and resistance protein-mediated defense.

### ECTOPIC EXPRESSION OF *CsNDR1* LEADS TO ACTIVATION OF SA-MEDIATED DEFENSE AND BROAD-SPECTRUM DISEASE RESISTANCE

Salicylic acid is a key signaling molecule regulating defense pathways including basal defense, R gene-mediated resistance, and systemic acquired resistance (Durrant and Dong, 2004; Lu, 2009). To test whether SA-mediated defense is activated in the transgenic plants, we quantified SA levels. We found that line 15 but not line 9 accumulated much higher levels of both free and total SA (glucosylated SA; **Figure 4C**). Consistent with its high SA levels, line 15 also showed higher expression of the SA marker gene *PR1* (**Figure 4D**). These results suggest that overexpression of *CsNDR1* to a certain level activates SA signaling.

**FIGURE 5:**
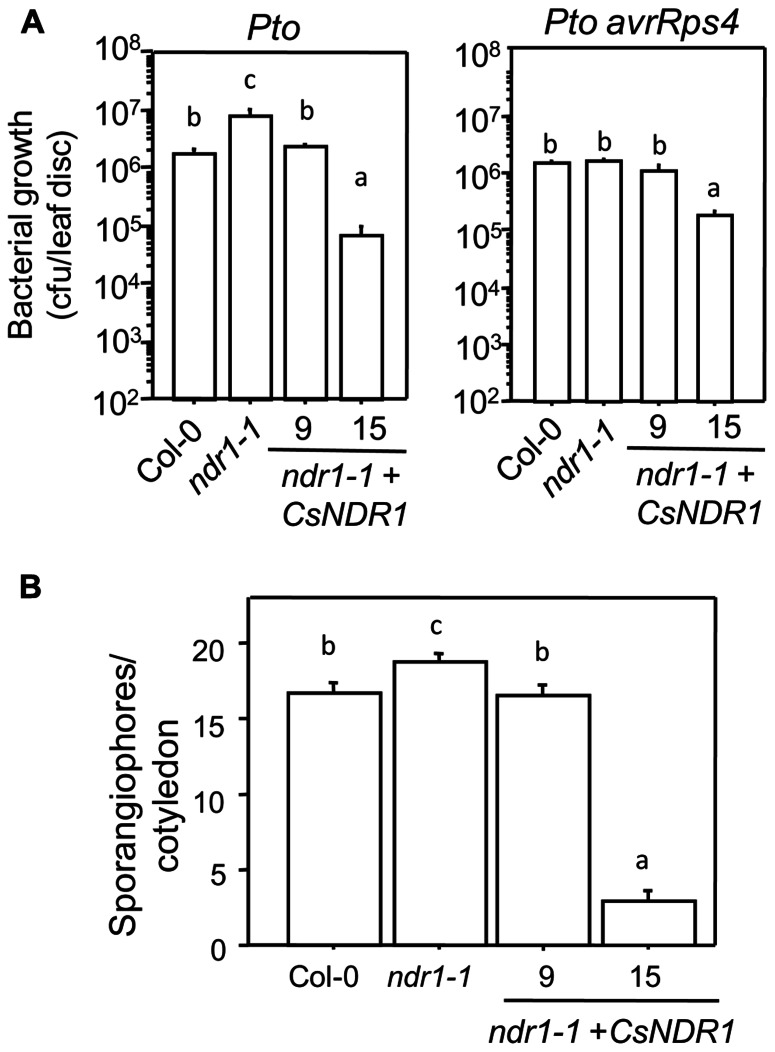
***CsNDR1 *confers broad-spectrum disease resistance. (A)** Bacterial growth assay. Twenty-five-day-old plants were infected with *Pto* (left panel; OD_600_ = 0.0002) or *Pto avrRps4* (right panel; OD_600_ = 0.0004). Bacterial growth was assessed 3 days after infection. Data represent the average of bacterial numbers in six samples ± standard error. **(B)** *Hpa* sporangiophore measurement. Seven-day-old seedlings were spray-infected with *Hpa* Noco2 (5 × 10^4^ spores/ml in water)*.* Statistical analysis was performed with one-way ANOVA Fisher’s PLSD tests (StatView 5.0.1). Different letters indicate significant difference among the samples (*P* < 0.05). These experiments were repeated twice with similar results.

To further investigate how overexpressing *CsNDR1* affects disease resistance, we challenged line 9 and 15 with additional *P. syringae* strains. Ectopic expression of *CsNDR1* complemented susceptibility conferred by *ndr1-1* to the virulent strain *P. syringae* pv. *tomato* DC3000 (*Pto*; **Figure 5A**, left). Line 15 is also more resistant to the isogenic avirulent strain *Pto avrRps4* (**Figure 5A**, right), which is recognized by RPS4 (a TIR–NBS–LRR type of R protein) independently of NDR1 (Aarts et al., 1998). Thus, these results indicate that ectopic expression of *CsNDR1* leads to activation of resistance to a pathogen that the endogenous gene otherwise does not have an effect on. In addition, we found that line 9 and 15 showed increased resistance to the virulent oomycete pathogen *Hpa* Noco2, compared with *ndr1-1* (**Figure 5B**). Thus, overexpressing *CsNDR1* could lead to broad-spectrum disease resistance.

### SA ACCUMULATION AND EXPRESSION OF *CsNDR1* ARE MODESTLY INDUCED BY *Ca*. L. asiaticus INFECTION

**FIGURE 6:**
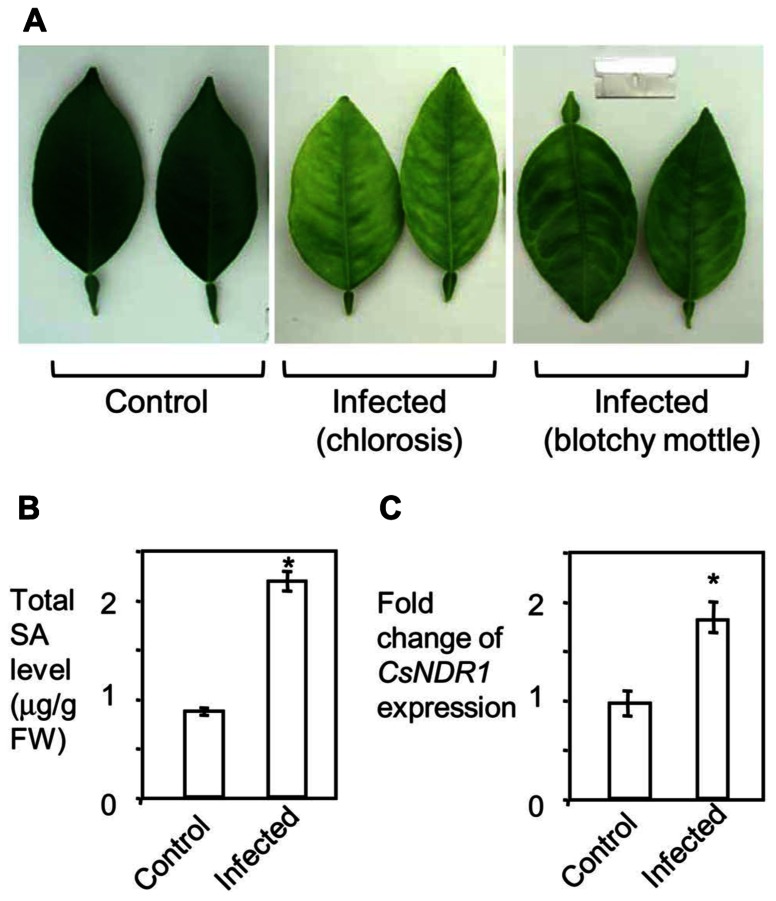
***Ca.* L. asiaticus infection induces modest increase of SA accumulation and *CsNDR1* expression in citrus. Fifteen-month-old “Valencia” plants were infected with *Ca.* L. asiaticus or mock treated. (A)** Photo of leaves collected at 16 wai. Note the infected leaves showing chlorosis and blotchy mottling, typical HLB symptoms. **(B)** SA quantitation. **(C)** qRT-PCR analysis. *CsNDR1* expression was normalized to the expression of *18S rRNA* and is presented as mean fold differences relative to control. Results are based on six biological replications. Statistical analysis was performed with one-way ANOVA Fisher’s PLSD tests (StatView 5.0.1). Asterisks indicate significant different between the samples (*P* < 0.05). These experiments were repeated two times with similar results.

To see how SA signaling is affected by HLB in citrus, we infected 15-month-old “Valencia” plants with *Ca.* L. asiaticus, using mock-treated plants as a control. We began to observe at 11 wai HLB symptoms, chlorosis and/or blotchy mottling of leaves, which increased in severity by 16 wai (**Figure 6A**). We did qPCR with the symptomatic and control leaves, using primers specific to *Ca.* L. asiaticus. The average Ct values of the symptomatic leaves were 21.8 at 11 wai and 20.7 at 16 wai. No *Ca.* L. asiaticus was detected in the control. Thus these symptomatic leaves were confirmed to be *Ca.* L. asiaticus positive. The symptomatic leaves were further collected for SA measurement and RNA analysis. Compared with the mock control, the symptomatic leaves from infected plants had about twofold more total SA levels (**Figure 6B**). Similarly, expression of *CsNDR1* was also induced about twofold more in the infected leaves (**Figure 6C**). These data suggest that SA and/or *CsNDR1*-mediated signaling are activated modestly upon *Ca.* L. asiaticus infection.

## DISCUSSION

In this study, we presented bioinformatic and experimental evidence suggesting that the citrus gene *CsNDR1* is an *Arabidopsis* *NDR1* ortholog. Overexpression of *CsNDR1* in *Arabidopsis* rescues *ndr1-1*-conferred susceptibility to *P. syringae *infection and leads to broad-spectrum disease resistance to the oomycete pathogen *Hpa* Noco2. We also found that both SA accumulation and *CsNDR1* expression are induced to modest levels in citrus upon infection with *Ca.* L. asiaticus, the agent associated with HLB. Our data suggest a possibility that manipulation of SA/*CsNDR1*-mediated defense may lead to enhanced resistance to HLB and other devastating diseases in citrus.

SA plays a critical role in regulating plant resistance against various pathogens. Broad-spectrum disease resistance has been successfully introduced into several economically important plants via manipulation of the SA pathway. Much of the previous studies have been focused on NPR1, a key SA signal transducer. For instance, overexpression of *Arabidopsis* NPR1 and/or its homologs from other plants confers resistance against diverse bacterial and fungal pathogens in *Arabidopsis*, apple, citrus, soybean, tomato, rice, and/or wheat (Fitzgerald et al., 2004; Li et al., 2004; Makandar et al., 2006; Malnoy et al., 2007; Sandhu et al., 2009; Zhang et al., 2010). These observations suggest that SA-mediated defense is conserved in monocots and dicots, and can be activated to against a range of pathogens (Campbell et al., 2002). Consistent with this notion, reports showed that exogenous application of SA agonists, such as benzothiadiazole and its commercial forms, acibenzolar-*S*-methyl (ASM, Actigard^®^, Syngenta Crop Protection, Inc.) and imidacloprid (Imid, Admire^®^, Bayer Crop Science), to citrus and other plants could induce defense marker gene expression and/or activate some protections of plants to a variety of viral, bacterial, and fungal pathogens (Campbell et al., 2002; Maxson-Stein et al., 2002; Dekkers et al., 2004; Francis et al., 2009; Graham and Myers, 2009).

*Arabidopsis* *NDR1* is a positive SA regulator, which is known to be specifically required for defense activated by some CC–NBS–LRR R proteins but not by some TIR–NBS–LRR type of R proteins (Aarts et al., 1998; Coppinger et al., 2004). Although not much is known about R-avr recognition in many non-*Arabidopsis* plants, the fact that *NDR1* homologs widely exist in diverse plants (Lee et al., 2006; Chong et al., 2008; Cacas et al., 2011) suggests conserved defense signaling involving *NDR1* homologs. Recently a coffee *NDR1* homolog was shown to complement the *ndr1-1* mutant for its susceptibility to *P. syringae* strains (Cacas et al., 2011). Here we also showed a complementation of *ndr1-1* by *CsNDR1*. The transgenic plants showed varying levels of *CsNDR1* expression, which is not uncommon for transgenes (Lu et al., 2003). We noticed that there is a degree of correlation between the level of transgene expression and the level of disease resistance in some transgenic plants (**Figure 2**). One line highly expressing *CsNDR1* (line 15) constitutively activates SA-mediated defense, associated with enhanced disease resistance to *Pto avrRps4* (that is not affected by the endogenous *Arabidopsis* NDR1) and to the oomycete isolate *Hpa Noco2* (**Figure 5**). It is known that signals induced by different R genes upon recognition of their cognate effectors from pathogens can converge at downstream steps, involving SA-mediated defense (Martin et al., 2003; Chisholm et al., 2006). Our data suggest that hyper-activation of one branch of R-gene pathways, such as the *CsNDR1* branch, could potentially activate SA signaling, leading to broad-spectrum disease resistance.

In *Arabidopsis*, both resistant and susceptible responses to pathogen infection are characterized by elevated SA accumulation and defense gene induction but with differences in the speed and amplitude of the responses (Zhou et al., 1998; Maleck et al., 2000; Tao et al., 2003; Song et al., 2004). Compared with a resistance response in *Arabidopsis*, induction of SA levels in citrus infected with *Ca.* L. asiaticus is quite small (**Figure 6B**). In addition, our gene expression data (**Figure 6C**) and microarray analyses (Albrecht and Bowman, 2008, 2012) indicate that the spectrum and intensity of defense genes induced by *Ca.* L. asiaticus are also quite limited. These observations suggest that when infected by *Ca.* L. asiaticus, citrus plants do not activate considerable host defense. This can be explained with at least two possible reasons: (1) a lack of recognition of effector proteins from *Ca.* L. asiaticus by citrus; and/or (2) a suppression of host defense by *Ca.* L. asiaticus. Thus, the interaction between citrus and *Ca.* L. asiaticus can be viewed as a compatible interaction, leading to disease symptom development in the host. The relatively low level of host defense in response to *Ca.* L. asiaticus also suggests a possibility that HLB resistance can be achieved if we could manipulate the host to enhance its defense levels.

 Genetic engineering is a particularly attractive approach to introduce disease resistance traits into citrus because citrus has long juvenile growth – it typically takes 5–15 years for a citrus plant to flower. In addition, most commercial citrus cultivars produce polyembryonic seeds asexually, which complicates the process of introducing novel traits into citrus via traditional breeding (Koltunow et al., 1996). Recently the *Arabidopsis* *NPR1* gene was shown to increase resistance to the canker disease when overexpressed in citrus (Zhang et al., 2010). Thus, *CsNDR1*, citrus *NPR1*, and other citrus homologs of SA regulatory genes are ideal candidates that can be genetically manipulated to increase their expression in order to test if these genes confer resistance to HLB in citrus. Engineering such genes could yield citrus plants with enhanced disease resistance that are also more acceptable to the consumers than those engineered with similar genes from other plants. Moreover, the newly released citrus genome sequence has greatly facilitated the identification of additional citrus defense genes. We anticipate that large-scale functional genomic analysis could uncover defense genes that play critical roles in resistance against HLB, citrus canker, and/or other emerging diseases challenging the citrus industry worldwide.

## Conflict of Interest Statement

The authors declare that the research was conducted in the absence of any commercial or financial relationships that could be construed as a potential conflict of interest.

## Footnotes

1 http://blast.ncbi.nlm.nih.gov/Blast.cgi

2 http://www.phytozome.net/search.php?method=Org_Csinensis

3 http://www.phytozome.net

## References

[B1] Aarts N. Metz M. Holub E. Staskawicz B. J. Daniels M. J. Parker J. E. (1998). Different requirements for *EDS1* and *NDR1* by disease resistance genes define at least two *R* gene-mediated signaling pathways in *Arabidopsis*. *Proc. Natl. Acad. Sci. U.S.A.* 95 10306–10311 9707643 10.1073/pnas.95.17.10306 PMC21504

[B2] Albrecht U. Bowman K. (2008). Gene expression in *Citrus sinensis* (L.) Osbeck following infection with the bacterial pathogen *Candidatus* Liberibacter asiaticus causing Huanglongbing in Florida. *Plant Sci.* 175 291–306

[B3] Albrecht U. Bowman K. D. (2012). Transcriptional response of susceptible and tolerant citrus to infection with *Candidatus* Liberibacter asiaticus. *Plant Sci.* 185–186 118–130 10.1016/j.plantsci.2011.09.008 22325873

[B4] Bittner-Eddy P. D. Beynon J. L. (2001). The *Arabidopsis* downy mildew resistance gene, *RPP13-Nd*, functions independently of *NDR1* and *EDS1* and does not require the accumulation of salicylic acid. *Mol. Plant Microbe Interact.* 14 416–421 11277440 10.1094/MPMI.2001.14.3.416

[B5] Bove J. M. (2006). Huanglongbing: a destructive, newly-emerging, century-old disease of citrus. *J. Plant Pathol.* 88 7–37

[B6] Brlansky R. H. Rogers M. E. (2007). Citrus Huanglongbing: understanding the vector–pathogen interaction for disease management. *Plant Health Progr.* 10.1094/APSnetFeature-2007-1207(publishedonline).

[B7] Cacas J. L. Petitot A. S. Bernier L. Estevan J. Conejero G. Mongrand S. (2011). Identification and characterization of the Non-race specific Disease Resistance 1 (NDR1) orthologous protein in coffee. *BMC Plant Biol. * 11:144 10.1186/1471-2229-11-144 PMC3212813 22023696

[B8] Campbell M. A. Fitzgerald H. A. Ronald P. C. (2002). Engineering pathogen resistance in crop plants. *Transgenic Res.* 11 599–613 12509135 10.1023/a:1021109509953

[B9] Cao H. Glazebrook J. Clarke J. D. Volko S. Dong X. (1997). The *Arabidopsis NPR1* gene that controls systemic acquired resistance encodes a novel protein containing ankyrin repeats. *Cell* 88 57–63 9019406 10.1016/s0092-8674(00)81858-9

[B10] Century K. S. Holub E. B. Staskawicz B. J. (1995). NDR1, a locus of *Arabidopsis thaliana* that is required for disease resistance to both a bacterial and a fungal pathogen. *Proc. Natl. Acad. Sci. U.S.A.* 92 6597–6601 11607554 10.1073/pnas.92.14.6597 PMC41565

[B11] Century K. S. Shapiro A. D. Repetti P. P. Dahlbeck D. Holub E. Staskawicz B. J. (1997). NDR1, a pathogen-induced component required for *Arabidopsis* disease resistance. *Science* 278 1963–1965 9395402 10.1126/science.278.5345.1963

[B12] Chern M. S. Fitzgerald H. A. Yadav R. C. Canlas P. E. Dong X. Ronald P. C. (2001). Evidence for a disease-resistance pathway in rice similar to the NPR1-mediated signaling pathway in *Arabidopsis*. *Plant J.* 27 101–113 11489188 10.1046/j.1365-313x.2001.01070.x

[B13] Chisholm S. T. Coaker G. Day B. Staskawicz B. J. (2006). Host–microbe interactions: shaping the evolution of the plant immune response. *Cell* 124 803–814 16497589 10.1016/j.cell.2006.02.008

[B14] Chomczynski P. Sacci N. (1987). Single-step method of RNA isolation by acid guanidinium thiocyanate–phenol–chloroform extraction. *Anal. Biochem.* 162 156–159 2440339 10.1006/abio.1987.9999

[B15] Chong J. Le Hananff G. Bertsch C. Walter B. (2008). Identification, expression analysis, and characterization of defense and signaling genes in *Vitis vinifera*. *Plant Physiol. Biochem.* 46 469–481 17988883 10.1016/j.plaphy.2007.09.010

[B16] Clough S. J. Bent A. F. (1998). Floral dip: a simplified method for *Agrobacterium*-mediated transformation of *Arabidopsis thaliana*. *Plant J.* 16 735–743 10069079 10.1046/j.1365-313x.1998.00343.x

[B17] Coppinger P. Repetti P. P. Day B. Dahlbeck D. Mehlert A. Staskawicz B. J. (2004). Overexpression of the plasma membrane-localized NDR1 protein results in enhanced bacterial disease resistance in *Arabidopsis thaliana*. *Plant J.* 40 225–237 15447649 10.1111/j.1365-313X.2004.02203.x

[B18] Dekkers M. G. H. Graham J. H. Burns J. K. Cubero J. Colburn G. C. (2004). Evaluation of chemical inducers and PR protein reporters for induced systemic resistance to citrus bacterial diseases. *Phytophathology* S25

[B19] Dong X. (2004). NPR1, all things considered. *Curr. Opin. Plant. Biol.* 7 547–552 15337097 10.1016/j.pbi.2004.07.005

[B20] Dormann P. Hoffman-Benning S. Balbo I. Benning C. (1995). Isolation and characterization of an *Arabidopsis* mutant deficient in the thylakoid lipid digalactosyl diacylglycerol. *Plant Cell* 7 1801–1810 8535135 10.1105/tpc.7.11.1801 PMC161039

[B21] Dörmanna P. Gopalanb S. He S.-Y. Benning C. (2000). A gene family in *Arabidopsis thaliana* with sequence similarity to *NDR1* and *HIN1*. *Plant Physiol. Biochem.* 38 789–796

[B22] Durrant W. E. Dong X. (2004). Systemic acquired resistance. *Annu. Rev. Phytopathol.* 42 185–209 15283665 10.1146/annurev.phyto.42.040803.140421

[B23] Falk A. Feys B. J. Frost L. N. Jones J. D. Daniels M. J. Parker J. E. (1999). EDS1, an essential component of *R* gene-mediated disease resistance in *Arabidopsis* has homology to eukaryotic lipases. *Proc. Natl. Acad. Sci. U.S.A.* 96 3292–3297 10077677 10.1073/pnas.96.6.3292 PMC15935

[B24] Fitzgerald H. A. Chern M. S. Navarre R. Ronald P. C. (2004). Overexpression of (*At*)*NPR1* in rice leads to a BTH- and environment-induced lesion-mimic/cell death phenotype. *Mol. Plant Microbe Interact.* 17 140–151 14964528 10.1094/MPMI.2004.17.2.140

[B25] Francis M. I. Redondo A. Burns J. K. Graham J. H. (2009). Soil application of imidacloprid and related SAR inducing compounds produces effective and persistent control of citrus canker. *Eur. J. Plant Pathol.* 124 283–292

[B26] Gao F. Shu X. Ali M. B. Howard S. Li N. Winterhagen P. (2010). A functional EDS1 ortholog is differentially regulated in powdery mildew resistant and susceptible grapevines and complements an *Arabidopsis eds1* mutant. *Planta* 231 1037–1047 20145949 10.1007/s00425-010-1107-z

[B27] Gaskalla R. (2006). *Comprehensive Report on Citrus Canker Eradication Program in Florida Through 14 January 2006 Revised*. Tallahassee, FL: Division of Plant Industry, Florida Department of Agriculture and Consumer Services, 25 p

[B28] Gottwald T. R. (2007). Citrus canker and citrus huanglongbing, two exotic bacterial diseases threatening the citrus industries of the western hemisphere. *Outlooks Pest Manage.* 18 274–279

[B29] Graham J. H. Myers M. (2009). Soil drenches of imidacloprid, thiamethoxam and acibenzolar-S-methyl for induction of SAR to control citrus canker in young citrus trees. *Phytopathology* 99 S46 10.1094/PDIS-09-10-0653 30731904

[B30] Hammond-Kosack K. E. Jones J. D. (1996). Resistance gene-dependent plant defense responses. *Plant Cell* 8 1773–1791 8914325 10.1105/tpc.8.10.1773 PMC161314

[B31] Ichinose K. Miyazi K. Matsuhira K. Yasuda K. Sadoyama Y. Do H. T. (2010). Unreliable pesticide control of the vector psyllid *Diaphorina citri* (Hemiptera: Psyllidae) for the reduction of microorganism disease transmission. *J. Environ. Sci. Health B* 45 466–472 20512737 10.1080/03601231003800263

[B32] Knepper C. Savory E. A. Day B. (2011). *Arabidopsis* NDR1 is an integrin-like protein with a role in fluid loss and plasma membrane-cell wall adhesion. *Plant Physiol.* 156 286–300 21398259 10.1104/pp.110.169656 PMC3091050

[B33] Koltunow A. M. Hidaka T. Robinson S. P. (1996). Polyembryony in citrus accumulation of seed storage proteins in seeds and in embryos cultured *in vitro*. *Plant Physiol.* 599–609 8742336 10.1104/pp.110.2.599 PMC157756

[B34] Lee M. W. Jelenska J. Greenberg J. T. (2008). *Arabidopsis* proteins important for modulating defense responses to *Pseudomonas syringae* that secrete HopW1-1. *Plant J.* 54 452–465 18266921 10.1111/j.1365-313X.2008.03439.x

[B35] Lee S. B. Ham B. K. Park J. M. Kim Y. J. Kh P. (2006). BnNHL18A shows a localization change by stress-inducing chemical treatments. *Biochem. Biophys. Res. Comm.* 339 399–406 16298336 10.1016/j.bbrc.2005.10.210

[B36] Li J. Brader G. Palva E. T. (2004). The WRKY70 transcription factor: a node of convergence for jasmonate-mediated and salicylate-mediated signals in plant defense. *Plant Cell* 16 319–331 14742872 10.1105/tpc.016980 PMC341906

[B37] Li W. Hartung J. S. Levy L. (2006). Quantitative real-time PCR for detection and identification of *Candidatus Liberibacter* species associated with citrus huanglongbing. *J. Microbiol. Methods* 66 104–115 16414133 10.1016/j.mimet.2005.10.018

[B38] Lin W. C. Lu C. F. Wu J. W. Cheng M. L. Lin Y. M. Yang N. S. (2004). Transgenic tomato plants expressing the *Arabidopsis NPR1* gene display enhanced resistance to a spectrum of fungal and bacterial diseases. *Transgenic Res.* 13 567–581 15672838 10.1007/s11248-004-2375-9

[B39] Livak K. J. Schmittgen T. D. (2001). Analysis of relative gene expression data using real-time quantitative PCR and the 2(-delta delta C(T)) method. *Methods* 25 402–408 11846609 10.1006/meth.2001.1262

[B40] Lu H. (2009). Dissection of salicylic acid-mediated defense signaling networks. *Plant Signal. Behav.* 4 713–717 19820324 10.4161/psb.4.8.9173 PMC2801381

[B41] Lu H. Rate D. N. Song J. T. Greenberg J. T. (2003). ACD6, a novel ankyrin protein, is a regulator and an effector of salicylic acid signaling in the *Arabidopsis* defense response. *Plant Cell* 15 2408–2420 14507999 10.1105/tpc.015412 PMC197305

[B42] Makandar R. Essig J. S. Schapaugh M. A. Trick H. N. Shah J. (2006). Genetically engineered resistance to *Fusarium* head blight in wheat by expression of *Arabidopsis* NPR1. *Mol. Plant Microbe Interact.* 19 123–129 16529374 10.1094/MPMI-19-0123

[B43] Malamy J. Carr J. P. Klessig D. F. Raskin I. (1990). Salicylic acid: a likely endogenous signal in the resistance response of tobacco to viral infection. *Science* 250 1002–1004 17746925 10.1126/science.250.4983.1002

[B44] Maleck K. Levine A. Eulgem T. Morgan A. Schmid J. Lawton K. A. (2000). The transcriptome of *Arabidopsis thaliana* during systemic acquired resistance. *Nat. Genet.* 26 403–410 11101835 10.1038/82521

[B45] Malnoy M. Jin Q. Borejsza-Wysocka E. E. He S. Y. Aldwinckle H. S. (2007). Overexpression of the apple *MpNPR1* gene confers increased disease resistance in *Malus*×*domestica*. *Mol. Plant Microbe Interact.* 20 1568–1580 17990964 10.1094/MPMI-20-12-1568

[B46] Martin G. B. Bogdanove A. J. Sessa G. (2003). Understanding the functions of plant disease resistance proteins. *Annu. Rev. Plant Biol.* 54 23–61 14502984 10.1146/annurev.arplant.54.031902.135035

[B47] Maxson-Stein K. He S. Hammerschmidt R. Jones A. (2002). Effect of treating apple trees with acibenzolar-S-methyl on fire blight and expression of pathogenesis-related protein genes. *Plant Disease* 86 785–790 10.1094/PDIS.2002.86.7.785 30818578

[B48] McDowell J. M. Cuzick A. Can C. Beynon J. Dangl J. L. Holub E. B. (2000). Downy mildew (*Peronospora parasitica*) resistance genes in *Arabidopsis* vary in functional requirements for NDR1, EDS1, NPR1 and salicylic acid accumulation. *Plant J.* 22 523–529 10886772 10.1046/j.1365-313x.2000.00771.x

[B49] McDowell J. M. Hoff T. Anderson R. G. Deegan D. (2010). Propagation, storage, and assays with *Hyaloperonospora arabidopsidis*: a model oomycete pathogen of *Arabidopsis*. *Methods Mol. Biol.* 712 137–151 21359806 10.1007/978-1-61737-998-7_12

[B50] McDowell J. M. Simon S. A. (2006). Recent insights into *R* gene evolution. *Mol. Plant Pathol.* 7 437–448 20507459 10.1111/j.1364-3703.2006.00342.x

[B51] Ng G. Seabolt S. Zhang C. Salimian S. Watkins T. A. Lu H. (2011). Genetic dissection of salicylic acid-mediated defense signaling networks in *Arabidopsis*. *Genetics* 189 851–859 21900271 10.1534/genetics.111.132332 PMC3213356

[B52] Nishimura M. T. Dangl J. L. (2010). *Arabidopsis* and the plant immune system. *Plant J.* 61 1053–1066 20409278 10.1111/j.1365-313X.2010.04131.x PMC2859471

[B53] Pegadaraju V. Louis J. Singh V. Reese J. C. Bautor J. Feys B. J. (2007). Phloem-based resistance to green peach aphid is controlled by *Arabidopsis* PHYTOALEXIN DEFICIENT4 without its signaling partner ENHANCED DISEASE SUSCEPTIBILITY1. *Plant J.* 52 332–341 17725549 10.1111/j.1365-313X.2007.03241.x

[B54] Rassmussen J. B. Hammerschmidt R. Zook M. N. (1991). Systemic induction of salicylic acid accumulation in cucumber after inoculation with *Pseudomonas syringae* pv. *syringae*. *Plant Physiol.* 97 1342–1347 16668554 10.1104/pp.97.4.1342 PMC1081169

[B55] Ryals J. Weymann K. Lawton K. Friedrich L. Ellis D. Steiner H. Y. (1997). The *Arabidopsis* NIM1 protein shows homology to the mammalian transcription factor inhibitor I kappa B. *Plant Cell* 9 425–439 9090885 10.1105/tpc.9.3.425 PMC156928

[B56] Sandhu D. Tasma I. M. Frasch R. Bhattacharyya M. K. (2009). Systemic acquired resistance in soybean is regulated by two proteins, orthologous to *Arabidopsis* NPR1. *BMC Plant Biol.* 9:105 10.1186/1471-2229-9-105 PMC2738679 19656407

[B57] Shah J. Tsui F. Klessig D. F. (1997). Characterization of a salicylic acid-insensitive mutant (*sai1*) of *Arabidopsis thaliana*, identified in a selective screen utilizing the SA-inducible expression of the *tms2* gene. *Mol. Plant Microbe Interact.* 10 69–78 9002272 10.1094/MPMI.1997.10.1.69

[B58] Shapiro A. D. Zhang C. (2001). The role of NDR1 in avirulence gene-directed signaling and control of programmed cell death in *Arabidopsis*. *Plant Physiol.* 127 1089–1101 11706189 PMC129278

[B59] Sharma Y. K. Davis K. R. (1997). The effects of ozone on antioxidant responses in plants. *Free Radic. Biol. Med.* 23 480–488 9214586 10.1016/s0891-5849(97)00108-1

[B60] Song J. T. Lu H. Mcdowell J. M. Greenberg J. T. (2004). A key role for ALD1 in activation of local and systemic defenses in *Arabidopsis*. *Plant J.* 40 200–212 15447647 10.1111/j.1365-313X.2004.02200.x

[B61] Strommer J. Gregerson R. Vayda M. (1993). “Isolation and characterization of plant mRNA,” in *Methods in Plant Molecular Biology and Biotechnology* eds Greenberg B. M. Glick B. R. (Boca Raton, FL: CRC Press Inc.) 49–65

[B62] Tamura K. Peterson D. Peterson N. Stecher G. Nei M. Kumar S. (2011). MEGA5: molecular evolutionary genetics analysis using maximum likelihood, evolutionary distance, and maximum parsimony methods. *Mol. Biol. Evol.* 28 2731–2739 21546353 10.1093/molbev/msr121 PMC3203626

[B63] Tao Y. Xie Z. Chen W. Glazebrook J. Chang H. S. Han B. (2003). Quantitative nature of *Arabidopsis* responses during compatible and incompatible interactions with the bacterial pathogen *Pseudomonas syringae*. *Plant Cell* 15 317–330 12566575 10.1105/tpc.007591 PMC141204

[B64] Tsuda K. Sato M. Glazebrook J. Cohen J. D. Katagiri F. (2008). Interplay between MAMP-triggered and SA-mediated defense responses. *Plant J.* 53 763–775 18005228 10.1111/j.1365-313X.2007.03369.x

[B65] Varet A. Hause B. Hause G. Scheel D. Lee J. (2003). The *Arabidopsis* *NHL3* gene encodes a plasma membrane protein and its overexpression correlates with increased resistance to *Pseudomonas syringae* pv. *tomato* DC3000. *Plant Physiol.* 132 2023–2033 12913158 10.1104/pp.103.020438 PMC181287

[B66] Varet A. Parker J. Tornero P. Nass N. Nurnberger T. Dangl J. L. (2002). *NHL25* and *NHL3*, two *NDR1*/*HIN1*-like genes in *Arabidopsis thaliana* with potential role(s) in plant defense. *Mol. Plant Microbe Interact.* 15 608–616 12059109 10.1094/MPMI.2002.15.6.608

[B67] Wang G. F. Seabolt S. Hamdoun S. Ng G. Park J. Lu H. (2011a). Multiple roles of WIN3 in regulating disease resistance, cell death, and flowering time in *Arabidopsis*. *Plant Physiol.* 156 1508–1519 21543726 10.1104/pp.111.176776 PMC3135961

[B68] Wang G. Y. Shi J. L. Ng G. Battle S. L. Zhang C. Lu H. (2011b). Circadian clock-regulated phosphate transporter PHT4;1 plays an important role in *Arabidopsis* defense. *Mol. Plant* 4 516–526 21447757 10.1093/mp/ssr016 PMC3988428

[B69] Wildermuth M. C. Dewdney J. Wu G. Ausubel F. M. (2001). Isochorismate synthase is required to synthesize salicylic acid for plant defence. *Nature* 414 562–565 11734859 10.1038/35107108

[B70] Xiao S. Ellwood S. Calis O. Patrick E. Li T. Coleman M. (2001). Broad-spectrum mildew resistance in *Arabidopsis thaliana* mediated by RPW8. *Science* 291 118–120 11141561 10.1126/science.291.5501.118

[B71] Zhang C. Gutsche A. T. Shapiro A. D. (2004). Feedback control of the *Arabidopsis* hypersensitive response. *Mol. Plant Microbe Interact.* 17 357–365 15077668 10.1094/MPMI.2004.17.4.357

[B72] Zhang X. Francis M. I. Dawson W. O. Graham J. H. Orbovic V. Triplett E. W. (2010). Over-expression of the *Arabidopsis NPR1* gene in citrus increases resistance to citrus canker. *Eur. J. Plant Pathol.* 128 91–100

[B73] Zheng M. S. Takahashi H. Miyazaki A. Hamamoto H. Shah J. Yamaguchi I. (2004). Up-regulation of *Arabidopsis thaliana* NHL10 in the hypersensitive response to Cucumber mosaic virus infection and in senescing leaves is controlled by signalling pathways that differ in salicylate involvement. *Planta* 218 740–750 14666423 10.1007/s00425-003-1169-2

[B74] Zhou N. Tootle T. L. Tsui F. Klessig D. F. Glazebrook J. (1998). PAD4 functions upstream from salicylic acid to control defense responses in *Arabidopsis*. *Plant Cell* 10 1021–1030 9634589 10.1105/tpc.10.6.1021 PMC144042

